# Variants in *CXCR4* associate with juvenile idiopathic arthritis susceptibility

**DOI:** 10.1186/s12881-016-0285-3

**Published:** 2016-03-22

**Authors:** Terri H. Finkel, Jin Li, Zhi Wei, Wei Wang, Haitao Zhang, Edward M. Behrens, Emma L. Reuschel, Sophie Limou, Carol Wise, Marilynn Punaro, Mara L. Becker, Jane E. Munro, Berit Flatø, Øystein Førre, Susan D. Thompson, Carl D. Langefeld, David N. Glass, Joseph T. Glessner, Cecilia E. Kim, Edward Frackelton, Debra K. Shivers, Kelly A. Thomas, Rosetta M. Chiavacci, Cuiping Hou, Kexiang Xu, James Snyder, Haijun Qiu, Frank Mentch, Kai Wang, Cheryl A. Winkler, Benedicte A. Lie, Justine A. Ellis, Hakon Hakonarson

**Affiliations:** Division of Rheumatology, The Children’s Hospital of Philadelphia, 19104 Philadelphia, PA USA; Department of Pediatrics, University of Pennsylvania School of Medicine, 19104 Philadelphia, PA USA; The Center for Applied Genomics, The Children’s Hospital of Philadelphia, 19104 Philadelphia, PA USA; Department of Computer Science, New Jersey Institute of Technology, 07102 New Jersey, NJ USA; Basic Research Laboratory, Center for Cancer Research, National Cancer Institute, Leidos Biomedical Research Inc., Frederick National Laboratory, 21702 Frederick, MD USA; Sarah M. and Charles E. Seay Center for Musculoskeletal Research, Texas Scottish Rite Hospital for Children, 75219 Dallas, TX USA; Division of Rheumatology, Texas Scottish Rite Hospital, 75219 Dallas, TX USA; Division of Rheumatology, Children’s Mercy- Kansas City, 64108 Kansas City, MO USA; Murdoch Childrens Research Institute, 3052 Parkville, VIC Australia; Paediatric Rheumatology Unit, Royal Children’s Hospital, 3052 Parkville, VIC Australia; Department of Rheumatology, Oslo University Hospital, Rikshospitalet, Norway; Cincinnati Children’s Hospital Medical Center, 45229 Cincinnati, OH USA; Center for Public Health Genomics and Department of Biostatistical Sciences, Wake Forest School of Medicine, 27157 Winston-Salem, NC USA; Department of Psychiatry and Behavioral Sciences, UUniversity of Southern California, 90089 Los Angeles, CA USA; Institute of Immunology, Oslo University Hospital, 0027 Rikshospitalet, Oslo Norway; Department of Paediatrics, University of Melbourne, 3010 Melbourne, Australia; Division of Human Genetics, The Children’s Hospital of Philadelphia, 19104 Philadelphia, PA USA; Present Address: Department of Pediatrics, Nemours Research Institute, Nemours Children’s Hospital, 32827 Orlando, FL USA

**Keywords:** Juvenile idiopathic arthritis, Genome-wide association study, CXCR4, Targeted resequencing

## Abstract

**Background:**

Juvenile idiopathic arthritis (JIA) is the most common chronic rheumatic disease among children, the etiology of which involves a strong genetic component, but much of the underlying genetic determinants still remain unknown. Our aim was to identify novel genetic variants that predispose to JIA.

**Methods:**

We performed a genome-wide association study (GWAS) and replication in a total of 1166 JIA cases and 9500 unrelated controls of European ancestry. Correlation of SNP genotype and gene expression was investigated. Then we conducted targeted resequencing of a candidate locus, among a subset of 480 cases and 480 controls. SUM test was performed to evaluate the association of the identified rare functional variants.

**Results:**

The *CXCR4* locus on 2q22.1 was found to be significantly associated with JIA, peaking at SNP rs953387. However, this result is subjected to subpopulation stratification within the subjects of European ancestry. After adjusting for principal components, nominal significant association remained (*p* < 10^−4^). Because of its interesting known function in immune regulation, we carried out further analyses to assess its relationship with JIA. Expression of *CXCR4* was correlated with *CXCR4* rs953387 genotypes in lymphoblastoid cell lines (*p* = 0.014) and T-cells (*p* = 0.0054). In addition, rare non-synonymous and stop-gain sequence variants in *CXCR4*, putatively damaging for CXCR4 function, were significantly enriched in JIA cases (*p* = 0.015).

**Conclusion:**

Our results suggest the association of *CXCR4* variants with JIA, implicating that this gene may be involved in the pathogenesis of autoimmune disease. However, because this locus is subjected to population stratification within the subjects of European ancestry, additional replication is still necessary for this locus to be considered a true risk locus for JIA. This cell-surface chemokine receptor has already been targeted in other diseases and may serve as a tractable therapeutic target for a specific subset of pediatric arthritis patients with additional replication and functional validation of the locus.

**Electronic supplementary material:**

The online version of this article (doi:10.1186/s12881-016-0285-3) contains supplementary material, which is available to authorized users.

## Background

Pediatric arthritis is the leading cause of acquired disability in children, afflicting about one in 1000 children worldwide [1, 2], all ethnicities and both genders, with onset as early as the first year of life. Classification schemes for pediatric arthritis are under evolution, akin to the recent classification changes for adult rheumatoid arthritis [3]; juvenile rheumatoid arthritis (JRA) is the term used historically in North America, while juvenile idiopathic arthritis (JIA) is the preferred name elsewhere, and is now used increasingly worldwide. JIA is defined as a group of chronic arthritides of unknown etiology, occurring in children from 0 to 16 years of age [4]. Morbidity associated with JIA can be life-long – with as many as 50 % of children with JIA entering adulthood with active disease [1, 2] – and represents a significant medical, financial, and emotional burden for patients, their families, and society. In the United States alone, arthritis and rheumatic diseases impact more than 46 million adults and 300,000 children, at a cost of $128 billion annually in direct and indirect medical costs [5–7]. Adults with JIA have lower rates of employment than matched controls, and health-related quality of life is diminished in adults with JIA, particularly in those with active disease [8]. Prompt recognition of the disease is important in preventing permanent disability; however, lack of specific confirmatory testing often delays diagnosis. The optimal management of JIA remains complicated and poorly defined, despite recent advances in therapy [1, 2].

The etiology of JIA is largely unknown. To our knowledge, there are no data supporting a major role for environmental exposures [9]. This does not preclude a role of the environment in the pathogenesis of JIA, but research to identify environmental risk factors is lacking. On the other hand, strong contribution of genetic components has been implicated from twin to family studies [10]: monozygotic twins have a concordance rate between 25 and 40 %; the calculated sibling recurrence risk ratio (λs = 15–30) is similar to that calculated for type 1 diabetes. Sibling pairs tend to show concordance for age of onset, subtype and course; and a subset of patients with JIA exhibits a heritable predisposition to develop this disease with an autosomal dominant pattern of inheritance.

Yet, in comparison to other autoimmune diseases of similar prevalence, the genetic etiology of JIA remains largely elusive. The major histocompatibility complex (MHC), in particular, the *HLA-DRB1* locus, has been established as having the strongest influence on susceptibility to JIA [11], contributing ~20 % of the proportion of sibling recurrent risk [12]. Non-MHC loci are important as well, with 16 loci now associated with JIA at genome-wide significance. Fourteen of these were identified for the first time by a recent Immunochip analysis [13], a hypothesis-driven approach that focused upon genes with known associations with immune disorders [14]. To comprehensively search for genes related to JIA and given that the pathophysiological mechanisms underlying JIA are unknown, we took an unbiased approach of genome-wide association study (GWAS) and performed replication studies in independent cohorts, including a total of 1166 cases and 9500 controls after quality control (QC) filtering. We subsequently performed targeted resequencing at identified candidate locus of *CXCR4* gene among a subset of 480 cases and 490 controls. Here we report that variants in gene *CXCR4* associate with JIA.

## Methods

### Participants

The JIA cases in our study were recruited from five sites in USA, Australia, and Norway: Texas Scottish Rite Hospital for Children (TSRHC; Dallas, Texas), Children’s Mercy Hospitals and Clinics (CMHC; Kansas City, Missouri), the Children's Hospital of Philadelphia (CHOP; Philadelphia, Pennsylvania), the Murdoch Childrens Research Institute (MCRI; Royal Children’s Hospital, Melbourne, Australia), and Oslo University Hospital (OUH; Oslo, Norway). (Table 1, Additional file 1: Table S1). A subset of subjects from these sites has been described previously [15–19]. JIA diagnosis was made according to the International League of Associations for Rheumatology (ILAR) revised criteria [4] and confirmed using the JIA Calculator^TM^ software (URLs) [20], an algorithm-based tool adapted from the ILAR criteria. All JIA cases were of age of onset <16 years old.

**Table 1:** Demographic and clinical characteristics of our JIA dataset

JIA Subtypes^A^	% female	Age (years)^B^
Oligoarthritis, persistent	73 %	5.9 (2.9,9.7)
Oligoarthritis, extended	82 %	3.95 (2.4,7.8)
Polyarthritis, RF negative	79 %	7.48 (3.2,11.3)
Polyarthritis, RF positive	95 %	13.5 (10.3,15.1)
Systemic arthritis	67 %	6.6 (3.2,11.1)
Enthesitis-related arthritis	40 %	11.9 (9.0,14.1)
Psoriatic arthritis	71 %	9.9 (7.0,13.2)
Undifferentiated arthritis	55 %	9.8 (4.5,13.8)
Total	68 %	8.3 (3.8,12.2)

^A^Revised ILAR criteria.

^B^Age (years): median (25 % percentile, 75 % percentile)

The clinical data of JIA case in the CHOP cohort were collected from the JIA Registry maintained within the CHOP Division of Rheumatology; clinical data of case samples from TSRHC, CMHC, MCRI, and OUH were drawn from medical records provided by the respective sites and stored in a de-identified database at the Center for Applied Genomics of the CHOP Research Institute.

The control subjects used are unrelated and disease-free children recruited within the CHOP Healthcare Network. Control subjects had no history of JIA or other chronic illnesses and were screened as negative for a diagnosis of autoimmune diseases, based on data from CHOP’s electronic health record and by intake questionnaires obtained by the recruiting staff from the Center for Applied Genomics. A total of 6500 pediatric controls passed stringent quality control (QC) filtering, as detailed below; post-QC, cases and controls were matched based on the multidimensional scaling (MDS) analysis [21, 22]. For OUH cohort, the 3000 well-characterized subjects from the Wellcome Trust Case–control Consortium (WTCCC) [21] were used as controls.

We combined TSRHC and CMHC samples to form the discovery cohort, and kept CHOP, MCRI and OUH cohorts as three independent replication cohorts.

### Ethics statement

The study was approved by the institutional review boards of TSRHC, CMHC, CHOP, MCRI, OUH, and CCHMC, and was compliant with HIPAA regulations. Parental written informed consent was obtained from all participants prior to inclusion in this study for the purpose of DNA collection and genotyping.

### Genotyping

All samples except those in the OUH replication cohort were genotyped using Illumina HumanHap550 BeadChip or the Human610-Quad arrays. The 530,000 SNPs that are overlapped by these two platforms were included in the study. Samples in the OUH replication cohort were genotyped using the Affymetrix GeneChip 500 K Mapping Array Set.

### Quality control

We employed the MDS algorithm implemented in PLINK to infer population structure, with the 924 individuals from the HapMap project as reference. Only samples of genetically inferred European ancestry were kept for further analysis. We then applied QC filters to exclude samples of poor genotyping quality prior to association analysis. A sample was excluded if the genotype call rate was <95 % or if the sample showed excess or deficient heterozygosity (inbreeding coefficient |F| > 0.1). Cryptic relatedness or erroneous duplicates were evaluated using pair-wise identity-by-descent estimation, and the sample with lower genotype call rate was removed from each identified relative pair. In our study, we also eliminated SNPs with genotype call rate <98 %, with minor allele frequency (MAF) <1 % in either cases or controls, or if there was significant departure from Hardy-Weinberg equilibrium (*p* < 0.0001). In the discovery cohort, a total of 518,907 genotyped SNPs passed QC and were included in analysis.

### Principal component analysis (PCA)

After the above sample and SNP QC, we conducted principal component analysis, trying to resolve relationship within-European samples. Using PLINK, we performed LD pruning and SNP exclusion so that only independent SNPs (r^2^ < 0.2) on the autosomes were kept for PCA. We then performed PCA using EIGENSTRAT [23]. MDS analysis was also performed with the independent SNPs using PLINK.

### Association analyses

For genotyped SNPs, association was tested by basic allelic test (chi-square test) and the odds ratio was calculated with respect to the minor allele using PLINK [22]. Logistic regression analyses were additionally performed, including the first 10 coordinates from the MDS analysis as covariates. Similar analyses were also performed including the first 10 principal components from the PCA analysis as covariates. Conditional association analysis was performed by including the genotype of the most associated SNP rs953387 as a covariate.

### Imputation

Imputation was carried out using software MACH 1.0 [24], with the reference panel of the HapMap CEU samples (HapMap release 22, NCBI build 36). The default two-step procedure was adopted for imputation. Imputed SNPs with MAF <0.01 in either cases or controls and SNPs with poor imputation quality (*r*^2^ < 0.3) were excluded from further analysis. We also zeroed out imputed genotypes with a posterior probability of <0.9.

### Meta-analysis

We performed meta-analysis using the inverse variance based method implemented in software METAL [25], which accounts for the direction of association relative to a consistent reference allele and adopts a fixed effect model. In this method, the effect size estimate of each cohort is weighted by its corresponding standard error. All meta-analyses comply with MOOSE guidelines (URLs).

### Targeted resequencing

We selected 480 patients with JIA and 480 controls without history of any autoimmune or inflammatory diseases, all of European ancestry based on the above MDS analysis. Samples were pooled in batches of 8 cases or 8 controls. One control pool was excluded for final analysis because it failed QC. Library preparation for targeted resequencing was performed according to the TruSeq (Illumina) sample-preparation protocol. DNA libraries were then hybridized to customized probes for capturing *CXCR4* with NimbleGen SeqCap EZ Choice Library (Roche NimbleGen). The captured region is chr2:136871907–136895725, including introns. *CXCR4*-enriched libraries were sequenced on the HiSeq 2000 (Illumina).

We performed sequence alignment using BWA against the reference human genome (UCSC hg19). We achieved ~320X coverage per pool or ~40X per individual. We performed variant calling using SNVer [26], a statistical tool designed for pooled sequencing data. We used ANNOVAR software [27] to annotate variants. Each pool has 2 × 8 = 16 haplotypes, so we estimated allele frequency by rounding X/K*16, where X is the number of reads carrying the alternative allele, and K is the total coverage.

### Statistical analysis for targeted sequencing

For the sequencing data, we employed the SUM test [28] for testing the association of the identified multiple non-synonymous variants. We computed the SUM association p value using the R package AssotesteR. Specifically, we used a permutation version of SUM, in order to prevent an inflated Type I error.

### Sanger sequencing

We performed Sanger sequencing to validate the rare non-synonymous and stop-gain sequence variants identified by targeted resequencing. Primers for all the five variants were designed using software Primer3 [29, 30] (URLs). Purification of PCR products was conducted using ExoSAP-it (USB, Affymetrix), and Sanger sequencing on both strands was performed using Big Dye Terminator Cycle Sequencing Kit v3.1 Kit (Applied Biosystems) with ABI 3130xl Genetic Analyzer (Applied Biosystems).

### eQTL analyses

To test association between SNP genotypes and gene expression quantified in immortalized B-lymphocytes and T-cells, we performed in silico analysis using publicly available data from genome-wide expression analysis of quantitative trait loci (eQTL) of the 270 individuals genotyped in the HapMap Project (including 30 Caucasian trios of Northern and Western European origin [CEU]) [31–34] and the 85 individuals of the GenCord project (a collection of cell lines from umbilical cords of individuals of Western European origin) [35]. Linear regression was used to test the association between gene expression and SNP genotypes under additive model [32]. SNP genotype was coded as 0, 1, and 2, corresponding to the counts of the minor allele in each genotype.

## Results

### Replication of known JIA susceptibility loci

We performed a genome-wide association study in the discovery cohort (TSRHC + CMHC) including 388 children with JIA and 2500 genetically matched controls of European ancestry with high-quality SNP array data (Table 1, Additional file 1: Table S1). Then three independent JIA cohorts (CHOP, MCRI, OUH) were analyzed for replication and followed by meta-analysis of all the cohorts in a total of 1166 cases and 9500 controls (Table 1, Additional file 1: Table S1). The age at onset, gender, and subtype distributions of all case cohorts are similar to those reported previously for JIA [1].

We focused on the phenotypic commonality amongst our patients, that is, chronic inflammation of the joints. We observed a strong genome-wide significant association at the *HLA* locus on 6p21 (Additional file 1: Table S2, Additional file 1: Figure S1), A total of 24 SNPs surpassed genome-wide significance threshold at this region in the discovery cohort. Furthermore, three loci which previously have been implicated in JIA and several other autoimmune diseases [10, 36, 37] – the *PTPN22* locus on 1p13, the *IL2RA* locus on 10p15, and the *ANTXR2* locus on 4q21.21 – were nominally associated with JIA (Additional file 1: Table S3). Replication of these known JIA susceptibility loci demonstrated the validity of our study.

### Novel association of *CXCR4* common variants

In addition to these known loci, we found a novel association signal at 2q22.1, with the most significant marker being rs953387 (*p* = 2.07 × 10^−10^; OR 0.59, 95 % CI 0.50–0.69). Six genotyped SNPs and ten additional imputed SNPs located on 2q22.1 of p value < 5 × 10^−8^ (Additional file 1: Table S4–S5, Additional file 1: Figure S1–S2) in the discovery cohort. Conditional on the top SNP rs953387, the significant association of the other SNPs were ablated, suggesting a single association signal at this locus. These SNPs were all nominally significant in each of our three independent replication cohorts. (Additional file 1: Table S4). Combined meta-analysis of all four cohorts indicated that four SNPs at the 2q22.1 locus were of p values < 5 × 10^−8^ (Additional file 1: Table S4).

However, as *CXCR4* is near a known stratified locus (nearby the lactase gene) [38], logistic regression analysis including the first ten coordinates from the MDS analysis as covariates brought the locus p value below genome-wide significance (top associated SNP rs1016269, P = 4.70 × 10^−5^). Similarly, using the first 10 principal components as the covariates in the logistic region association, we obtained similar results (top associated SNP rs1016269, *P* = 2.80 × 10^−5^). Thus, our original association results were inflated due to subpopulation stratification within the subjects of European ancestry. The association of *CXCR4* was nominally significant with the best *p*-value <0.0001. When we evaluated the association by another method, permutation tests with the inclusion of the first ten coordinates from the MDS analysis as covariates in each association analysis, we obtained similar results of nominally significant results based on 10,000 permutations. *CXCR4* is an interesting gene because of its known role in immune regulation in a variety of immune cell types as a chemokine receptor. Gene *CXCR4*, is expressed on the surface of T-cells, B-cells, monocytes, neutrophils and dendritic cells (Additional file 1: Figure S3). The important function of CXCR4 during B cell development and activation [39, 40], and the key role of B cells in pathogenesis of JIA [41] make *CXCR4* an intriguing target worth of further examination. Because of sub-population stratification, additional replication is still necessary for this locus to be considered a true risk locus for JIA. Its association with JIA need to be further investigated by stratified analysis in more refined homogeneous sub-populations of European ancestry, with a larger sample size. Further functional studies would also be required to demonstrate its potential contribution to JIA etiology.

### Correlation between SNP genotype and *CXCR4* expression

Additionally, rs953387 was significantly associated with the mRNA levels of *CXCR4* (*p* = 0.014; Additional file 1: Figure S4) among HapMap dataset of 30 CEU children in the Sanger Institute GENe Expression VARiation (Genevar) public database (URLs), which profiles gene expression in immortalized B-lymphocyte samples [31]. Analysis of an independent dataset of T-cell lines from umbilical cords of 75 individuals of Western European origin [35] also demonstrated association between genotypes of another significant SNP rs1016269 at *CXCR4* locus (Additional file 1: Table S5) and levels of *CXCR4* expression (*p* = 0.0054; Additional file 1: Figure S3). These data suggest that common variants at *CXCR4* locus may regulate mRNA expression of this chemokine receptor.

### Novel association of CXCR4 rare variants

In addition to common variants, rare variants also contribute to disease susceptibity and usually confer a larger effect than common variants. We further examined rare variants at this locus by conducting deep resequencing in 480 JIA cases and 480 controls. Table 2 shows the variants identified in the coding region of *CXCR4*. Of interest are 4 non-synonymous and 1 stop-gain SNVs. These are all rare variants that are not catalogued in current variant databases (1000G and ESP6500) or with an extreme low frequency (0.0003). Four of these five rare variants are present only in JIA patients, not in controls. The SUM test produced a significance p value of 0.015 when testing the association of these 5 variants, suggesting that rare variants in the *CXCR4* gene may be contributing to the pathogenesis of JIA.

**Table 2:** Summary of variants in *CXCR4* coding-region by deep resequencing

Chr	Pos	Ref	Obs	Exonic Function	ESP6500	1000G	dbSNP135	CASE Freq.	CTRL Freq.
chr2	136872553	G	A	synonymous SNV	0.000461	0.0014	rs144110709	0	1
chr2	136872565	G	C	synonymous SNV				0	1
chr2	136872705	T	C	nonsynonymous SNV				0	1
chr2	136872715	G	A	synonymous SNV	0.003306	0.0018	rs148279552	3	7
chr2	136872727	A	G	synonymous SNV	0.000077		rs146627075	1	0
chr2	136872970	G	T	nonsynonymous SNV				1	0
chr2	136873084	G	A	synonymous SNV	0.036983	0.06	rs2228014	40	41
chr2	136873341	T	G	nonsynonymous SNV	0.000308		rs56400844	2	0
chr2	136873491	G	C	nonsynonymous SNV				1	0
chr2	136873496	A	T	stopgain SNV				1	0

*Chr* chromosome, *Pos* position on human genome build hg19, *Ref* reference allele, *Obs* the variant allele observed in our JIA dataset, *ESP6500* variant allele frequency in Exome Sequencing Project, *1000G* variant allele frequency in Thousand Genome Project, *dbSNP 135* SNP rs ID in Single Nucleotide Polymorphism database of human genome build 135, *CASE Freq* variant allele frequency among cases, *CTRL freq* variant allele frequency among controls

**Fig. 1:**
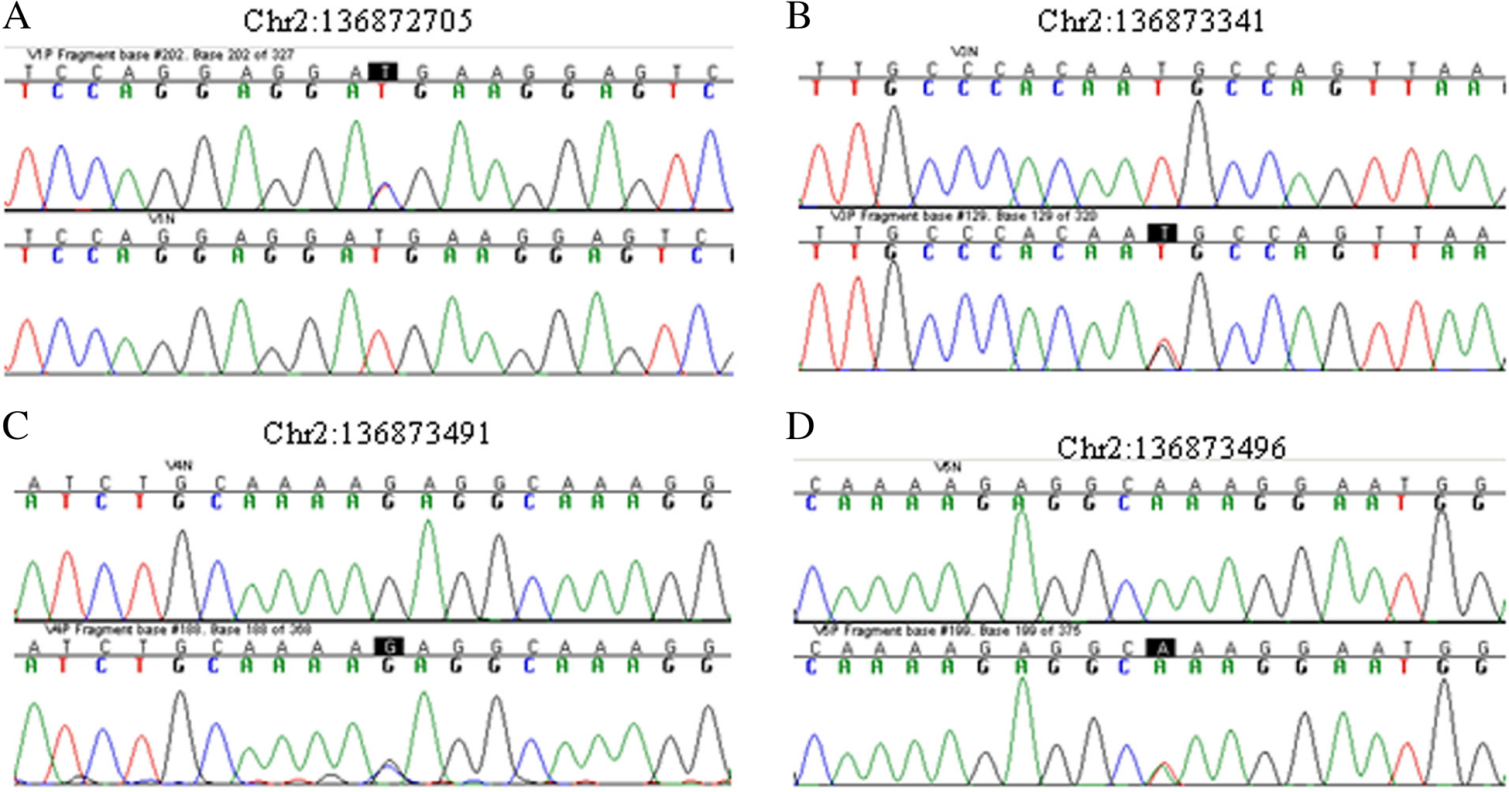
Sanger validation of rare variants in gene *CXCR4*. The sequencing chromatograms are shown for the sample carrying the rare variant allele and a representative control that does not have the rare variant allele. **a** chr2:136872705; **b** chr2:136873341; **c** chr2:136873491; **d** chr2:136873496. The rare variants are highlighted in the figure

Then we performed Sanger sequencing to validate their genotype status. The primer sequences for each rare variant are shown in Additional file 1: Table S6. Among 40 samples of the 5 pools from which the rare variants were detected, 33 samples were left with enough DNA for Sanger sequencing. No genomic DNA was left for 4 samples in pool from which variant chr2:136872970 was identified and 3 samples in pool from which variant chr2: 136873341 was found. Among the 33 samples with enough genomic DNA, 4 samples were found carrying the rare variant identified in deep sequencing (Fig. 1).

## Discussion

In a combined sample set of more than 1100 JIA subjects of European ancestry, we replicated common genetic variants at loci of *HLA*, *PTPN22*, *IL2RA* and *ANTXR2* that have been reported for association with susceptibility to JIA. We also found a nominally significant association at *CXCR4* which has been implicated in immune regulation, demonstrated the correlation between *CXCR4* variants genotype and its gene expression level. We further showed that rare non-synonymous and stop-gain variants in *CXCR4* are enriched in JIA cases. Our data support a role for altered expression of *CXCR4* in JIA pathogenesis, and present the first genetic demonstration of a potential role for the chemokine receptor, CXCR4, in the pathogenesis of autoimmune disease. Because this locus is subjected to population stratification within the subjects of European ancestry, additional replication is still necessary for this locus to be considered a true risk locus for JIA.

Few genetic studies of JIA have been carried out at genome-wide scale. Previously reported GWASs of JIA were all limited by small size [42, 43]. A more recent study using Immunochip, took a hypothesis-driven approach, targeting immune-specific genes. Several significant new loci were identified from the study [13], however the Immunochip does not capture variants across a large proportion of the genome. Our study was composed of a large dataset with comprehensive genome SNP coverage, and the candidate gene identified was examined using targeted resequencing with a large number of samples and high coverage.

Chemokine receptors impact immune system development and function in part via regulation of cell migration. The G protein-coupled chemokine receptor, *CXCR4*, is expressed on the surface of T-cells, B-cells, monocytes, neutrophils and dendritic cells (Additional file 1: Figure S3), and is activated exclusively by CXCL12 (also known as stromal-derived-factor-1, SDF-1), a small peptide mediator and potent chemoattractant for leukocytes, including B- and T-cells. CXCR4 and its ligand, CXCL12, have been shown to play a role in B-cell production, myelopoiesis, integrin activation, angiogenesis, and chemotaxis [25]. Intriguingly, the human immunodeficiency virus (HIV-1) has usurped CXCR4’s unique CXCL12 binding site, exploiting CXCR4 as a co-receptor in later stages of HIV-1 infection, and CXCR4 antagonists have been explored as treatments for HIV infection. Binding of CXCR4 to CXCL12 is also proposed to play a role in cancer metastases, and CXCR4 antagonists are under study in human clinical trials for solid and non-solid tumors [44]. Available therapeutic agents targeting the CXCR4-CXCL12 axis for activation or inhibition include plerixafor (AMD3100), recombinant CXCL12, and high-affinity CXCR4 and CXCL12 monoclonal antibodies, some of which are already in use in the clinic but not approved for use in children. The recent report of crystal structures of CXCR4 with small-molecule and cyclic peptide inhibitors [45] provide new opportunities for drug discovery efforts targeting this receptor.

CXCR4 and CXCL12 have been implicated in the pathogenesis of autoimmune diseases [44, 46]. In mouse models of autoimmune disease, modulation of CXCR4 alters trafficking of leukocytes to peripheral organs and polarization of regulatory T cells, and accelerates onset of disease [47, 48]. This is consistent with our data showing that a risk variant of *CXCR4* correlates with decreased expression of CXCR4. An alternate hypothesis is that the effect of low CXCR4 expression is indirect and leads to a compensatory increase of the CXCR4 ligand, CXCL12. Our preliminary data suggest that the risk variant of *CXCR4* correlates with increased expression of CXCL12 (data not shown). This is consistent with models of collagen-induced arthritis in which CXCL12 acts as a pro-inflammatory factor in the pathogenesis of inflammatory arthritis [49, 50], and with human studies in which CXCL12 enhances cellular proliferation and cytokine expression by peripheral blood T cells, upregulates expression of cytokines and chemokines by fibroblast-like synoviocytes from patients with RA [51], and mediates lymphocyte ingress into RA synovial tissue, synovial neovascularisation, and osteoclastogenesis [52]. Translation of our discovery into therapeutic benefit for a specific group of JIA patients will require additional genetic replication studies, proper functional validation and greater insight into the pathogenic role of CXCR4 in JIA.

## Conclusions

We uncovered common SNPs at the *CXCR4* locus associated with JIA and further found rare functional variants at *CXCR4* enriched in JIA cases. Our results suggest that genetic variants at the *CXCR4* locus may predispose to the development of JIA. Because of sub-population stratification, additional replication is still necessary for this locus to be considered a true risk locus for JIA. The extensive literature surrounding the biology of CXCR4 and its binding partner CXCL12 in health and disease, and the ready availability of targeted therapeutic agents make *CXCR4* a particularly attractive candidate for further genetic replication and functional investigation of its pathogenic role and therapeutic potential in JIA and other autoimmune diseases affecting children.

## Additional file

Additional file 1: Supplemental Data: Table S1. The clinical characteristics of samples in each JIA cohort. Table S2. Genome-wide significant associations at the *HLA* locus (*p* < 5×10^-8^ in the discovery cohort).Table S3. Association results for top SNPs in known JIA associated genes *PTPN22*, *IL2RA*, *ANTXR2*. TableS4. The most significantly associated SNPs at CXCR4 locus on chromosome 2q22.1. Table S5 . Genomewideassociation results for imputed SNPs (*p* < 1×10^-4^ in combined analysis) in the vicinity of *CXCR4* in our JIA cohort. Table S6. Primers used in Sanger sequencing validation of rare variants at *CXCR4* locus. Figure S1. Genome-wide association results for JIA. Figure S2. Regional association plot for the 2q22.1 region. Figure S3. *CXCR4* tissue-specific gene expression levels. Figure S4. *CXCR4* expression levels stratified by SNP genotype. (DOC 466 kb)
