# Supplementary material for: Variants in CXCR4 associate with juvenile idiopathic arthritis susceptibility
Source: BMC Med Genet. 2016 Mar 22;17:24. doi: 10.1186/s12881-016-0285-3 (PMC4804485; doi:10.1186/s12881-016-0285-3)
Supplement: Additional file 1: — Supplemental Data: Table S1. The clinical characteristics of samples in each JIA cohort. Table S2. Genome-wide significant associations at the HLA locus (p < 5×10^-8^ in the discovery cohort).Table S3. Association results for top SNPs in known JIA associated genes PTPN22, IL2RA, ANTXR2. TableS4. The most significantly associated SNPs at CXCR4 locus on chromosome 2q22.1. Table S5 . Genomewideassociation results for imputed SNPs (p < 1×10^-4^ in combined analysis) in the vicinity of CXCR4 in our JIA cohort. Table S6. Primers used in Sanger sequencing validation of rare variants at CXCR4 locus. Figure S1. Genome-wide association results for JIA. Figure S2. Regional association plot for the 2q22.1 region. Figure S3. CXCR4 tissue-specific gene expression levels. Figure S4. CXCR4 expression levels stratified by SNP genotype. (DOC 466 kb) [file 12881_2016_285_MOESM1_ESM.doc]

**Supplemental Data**

**Variants in *CXCR4* associate with Juvenile Idiopathic Arthritis susceptibility**

Terri H. Finkel, Jin Li, Zhi Wei, Wei Wang, Haitao Zhang, Edward M. Behrens, Emma L. Reuschel, Sophie M. Limou, Carol Wise, Marilynn Punaro, Mara L. Becker, Jane E. Munro, Berit Flatø, Øystein Førre, Susan D. Thompson, Carl D. Langefeld, David N. Glass, Joseph T. Glessner, Cecilia E. Kim, Edward Frackelton, Debra K. Shivers, Kelly A. Thomas, Rosetta M. Chiavacci, Cuiping Hou, Kexiang Xu, James Snyder, Haijun Qiu, Frank Mentch, Kai Wang, Cheryl A. Winkler, Benedicte A. Lie, Justine A. Ellis & Hakon Hakonarson

**Supplemental Table 1. The clinical characteristics of samples in each JIA cohort.** The discovery and replication cohorts of JIA following standard quality control and exclusion of subjects of non-European ancestry are shown.

| **JIA Subtypes^A^** | **Discovery^B^**  **(TSRHC+CMHC)** | **Replication 1**  **(CHOP)** | **Replication 2**  **(MCRI)** | **Replication 3**  **(OUH)** | **Total** |
| --- | --- | --- | --- | --- | --- |
| Oligoarthritis, persistent + extended | 135 | 45 | 76 | 181 | 437 |
| Polyarthritis, RF negative | 86 | 35 | 40 | 88 | 249 |
| Oligoarthritis+Polyarthritis, RF negative | 221 | 80 | 116 | 269 | 686 |
| Polyarthritis, RF positive | 19 | 7 | 10 | 18 | 54 |
| Polyarthritis, RF unknown | 27 | 0 | 0 | 0 | 27 |
| Systemic arthritis | 44 | 23 | 12 | 24 | 103 |
| Enthesitis-related arthritis | 38 | 32 | 6 | 62 | 138 |
| Psoriatic arthritis | 32 | 19 | 5 | 31 | 87 |
| Undifferentiated arthritis | 7 | 21 | 5 | 38 | 71 |
| Total number of JIA cases | 388 | 182 | 154 | 442 | 1166 |
| Total number of controls | 2500 | 2000 | 2000 | 3000 | 9500 |

^A^Revised ILAR criteria (1). For this study, a patient was considered to be rheumatoid factor (RF)-negative based on a single test; patients for whom the RF was indeterminant (27 patients) were excluded from subtype analysis.

^B^JIA Data Set: TSRHC; Texas Scottish Rite Hospital for Children, Dallas, Texas, USA; CMHC; Children’s Mercy Hospitals and Clinics, Kansas City, Missouri, USA; CHOP; The Children’s Hospital of Philadelphia, Philadelphia, Pennsylvania, USA; MCRI; Murdoch Childrens Research Institute, Melbourne, Australia; OUH; Department of Rheumatology, Oslo University Hospital, Rikshospitalet, Oslo, Norway.

**Supplemental Table 2.** **Genome-wide significant associations at the *HLA* locus (p<5×10^-8^ in the discovery cohort).**

| **Chr** | **SNP** | **Pos** | **Gene** | **Minor/Major Allele** | **Case_MAF** | **Control_MAF** | **OR** | **p value (Dis)** | **p value (Meta)** |
| --- | --- | --- | --- | --- | --- | --- | --- | --- | --- |
| 6 | rs2395148 | 32429532 | C6orf10 | T/G | 0.095 | 0.025 | 4.08 | 2.63E-23 | 7.95E-38 |
| 6 | rs2073048 | 32443411 | C6orf10 | T/C | 0.242 | 0.119 | 2.36 | 1.31E-20 | 3.43E-26 |
| 6 | rs7770048 | 32442732 | C6orf10 | T/C | 0.242 | 0.119 | 2.36 | 1.51E-20 | 1.86E-26 |
| 6 | rs4248166 | 32474399 | BTNL2 | C/T | 0.296 | 0.16 | 2.21 | 3.03E-20 | 3.19E-26 |
| 6 | rs2294884 | 32475237 | BTNL2 | C/A | 0.299 | 0.164 | 2.18 | 8.38E-20 | 4.19E-25 |
| 6 | rs13192471 | 32779081 | near HLA-DQB1 | C/T | 0.244 | 0.136 | 2.05 | 6.32E-15 | 3.70E-23 |
| 6 | rs6907322 | 32432923 | C6orf10 | A/G | 0.295 | 0.177 | 1.95 | 7.60E-15 | 1.78E-18 |
| 6 | rs1794275 | 32779226 | near HLA-DQB1 | T/C | 0.275 | 0.163 | 1.94 | 4.99E-14 | 4.80E-16 |
| 6 | rs9268365 | 32441417 | C6orf10 | T/G | 0.296 | 0.181 | 1.91 | 5.06E-14 | 4.41E-18 |
| 6 | rs10947262 | 32481290 | BTNL2 | T/C | 0.174 | 0.088 | 2.18 | 9.91E-14 | 1.84E-18 |
| 6 | rs7765379 | 32788906 | near HLA-DQA2 | G/T | 0.188 | 0.098 | 2.13 | 9.91E-14 | 8.27E-16 |
| 6 | rs3763313 | 32484449 | near BTNL2 | C/A | 0.284 | 0.18 | 1.8 | 1.29E-11 | 1.60E-14 |
| 6 | rs2071286 | 32287874 | NOTCH4 | A/G | 0.331 | 0.222 | 1.74 | 2.30E-11 | 2.17E-14 |
| 6 | rs1265048 | 31189388 | near C6orf15 | G/A | 0.457 | 0.338 | 1.65 | 9.57E-11 | 1.62E-10 |
| 6 | rs1035798 | 32259200 | AGER | T/C | 0.37 | 0.261 | 1.67 | 2.31E-10 | 1.76E-16 |
| 6 | rs2395185 | 32541145 | near HLA-DRA | T/G | 0.197 | 0.306 | 0.56 | 5.74E-10 | 3.27E-18 |
| 6 | rs411326 | 32319295 | near NOTCH4 | A/G | 0.342 | 0.244 | 1.61 | 7.39E-09 | 2.97E-06 |
| 6 | rs2516049 | 32678378 | near HLA-DRB1 | G/A | 0.196 | 0.295 | 0.58 | 1.05E-08 | 1.20E-19 |
| 6 | rs477515 | 32677669 | near HLA-DRB1 | T/C | 0.196 | 0.295 | 0.58 | 1.20E-08 | 1.46E-19 |
| 6 | rs2301226 | 33142574 | HLA-DPA1 | T/C | 0.214 | 0.137 | 1.72 | 1.52E-08 | 1.40E-11 |
| 6 | rs17576984 | 32320963 | near NOTCH4 | T/C | 0.148 | 0.085 | 1.87 | 1.84E-08 | 1.72E-13 |
| 6 | rs570963 | 32397572 | C6orf10 | C/T | 0.175 | 0.107 | 1.78 | 3.09E-08 | 5.09E-11 |
| 6 | rs3868075 | 31275794 | HCG27 | C/T | 0.501 | 0.397 | 1.52 | 4.40E-08 | 2.23E-04 |
| 6 | rs4713447 | 31270942 | near HCG27 | G/A | 0.501 | 0.397 | 1.52 | 4.45E-08 | 2.28E-04 |

Chr: Chromosome; Pos: Position in base pair on genome build hg19; Gene: The closest gene to the SNP; MAF, Minor allele frequencies in discovery cohort; OR, odds ratio in discovery cohort. The OR is calculated with respect to the minor allele.; p value (Dis), association test p value in discovery cohort; p value (meta), p value (meta): meta-analysis p value.

**Supplemental Table 3. Association results for top SNPs in known JIA associated genes *PTPN22*, *IL2RA*, *ANTXR2***

| SNP | CHR | BP | Gene | Minor/Major Alleles | Case_MAF | Control_MAF | OR | 95% CI | p value (Discovery) |
| --- | --- | --- | --- | --- | --- | --- | --- | --- | --- |
| rs2476601 | 1 | 114179091 | *PTPN22* | A/G | 0.1 | 0.085 | 1.65 | 1.31, 2.07 | 1.77E-05 |
| rs17509015 | 4 | 80885891 | *ANTXR2* | T/C | 0.13 | 0.17 | 0.72 | 0.58, 0.90 | 0.00434 |
| rs706779 | 10 | 6138830 | *IL2RA* | G/A | 0.43 | 0.48 | 0.84 | 0.72, 0.97 | 0.0217 |

BP, base pair chromosome coordinates; MAF, minor allele frequencies in discovery cohort; p value, basic allelic test p value; OR, odds ratio in discovery cohort; p value (combined), meta-analysis p value in the three cohorts typed genome-wide (discovery and replication cohorts 1 and 2).

**Supplemental Table 4**. **The most significantly associated SNPs at *CXCR4* locus on chromosome 2q22.1.**

| **CHR** | **SNP** | | **Position^A^ (NCBI 36)** | | **Minor/ Major Alleles^A^** | **Discovery (TSRHC+CMHC)** | | | | | **Replication 1 (CHOP)** | | | **Replication 2 (MCRI)** | | | **Replication 3 (OUH)** | | | **p value (Meta-analysis)** | **Het Pvalue (Meta-analysis)** |
| --- | --- | --- | --- | --- | --- | --- | --- | --- | --- | --- | --- | --- | --- | --- | --- | --- | --- | --- | --- | --- | --- |
|  |  | |  | |  | Minor allele frequency | | p value | OR^B^ | 95% CI | Minor allele frequency | | p value | Minor allele frequency | | p value | Minor allele frequency | | p value |  |  |
|  |  | |  | |  |  |  |  |  |  |  |  |  |  |  |  |  |  |  | Cases (N=1166) Controls (N=9500) |  |
|  |  | |  | |  | Case (N=388) | Control (N=2500) |  |  |  | Case (N=182) | Control (N=2000) |  | Case (N=154) | Control (N=2000) |  | Case (N=442) | Control (N=3000) |  |  |  |
| 2 | rs953387 | | 136623640 | | G/T | 0.29 | 0.41 | 2.07E-10 | 0.59 | 0.50-0.69 | 0.35 | 0.41 | 0.01 | 0.35 | 0.42 | 0.02 | 0.21 | 0.26 | 5.52E-04 | 2.78E-14 | 0.16 |
| 2 | rs1123848 | | 136661499 | | T/C | 0.28 | 0.39 | 3.89E-10 | 0.59 | 0.50-0.70 | 0.35 | 0.40 | 0.04 | 0.33 | 0.40 | 0.01 | NA | NA | NA | 1.85E-11 | 0.10 |
| 2 | rs4954564 | | 136611978 | | G/A | 0.30 | 0.41 | 3.08E-09 | 0.61 | 0.52-0.72 | 0.35 | 0.42 | 0.01 | 0.35 | 0.42 | 0.02 | 0.21 | 0.26 | 7.16E-04 | 1.26E-13 | 0.31 |
| 2 | rs10221893 | | 136730076 | | C/T | 0.39 | 0.51 | 4.58E-09 | 0.63 | 0.54-0.74 | 0.50 | 0.53 | 0.31 | 0.46 | 0.52 | 0.05 | 0.31 | 0.33 | 3.23E-01 | 2.39E-07 | 0.0045 |
| 2 | rs6430612 | | 136722668 | | C/T | 0.39 | 0.51 | 7.98E-09 | 0.64 | 0.55-0.74 | 0.49 | 0.53 | 0.17 | 0.46 | 0.52 | 0.05 | 0.31 | 0.33 | 3.23E-01 | 1.69E-07 | 0.0063 |
| 2 | rs1016269 | | 136657132 | | A/G | 0.15 | 0.24 | 4.01E-08 | 0.56 | 0.46-0.69 | 0.19 | 0.25 | 0.02 | 0.21 | 0.25 | 0.11 | 0.12 | 0.16 | 4.91E-03 | 2.37E-10 | 0.17 |
|  | |  | |  |  |  |  |  |  |  |  |  |  |  |  |  |  |  |  |  |  |
|  | | | |  |  |  |  |  |  |  |  |  |  |  |  |  |  |  |  |  |  |
|  | | | |  |  |  |  |  |  |  |  |  |  |  |  |  |  |  |  |  |  |
|  | | | | |  |  |  |  |  |  |  |  |  |  |  |  |  |  |  |  |  |
|  | | | | |  |  |  |  |  |  |  |  |  |  |  |  |  |  |  |  |  |

| p value, basic allelic test p value; OR, odds ratio; CI, confidence interval; p value (combined), meta-analysis p value in all 4 cohorts. | | | | |
| --- | --- | --- | --- | --- |
| ^A^ The chromosome coordinates and allele designations are on the basis of the forward strand of the NCBI 36 genome assembly. |  |  |  |  |
| ^B^ The odds ratio is calculated with respect to the minor allele. | | | | |

**Supplemental Table 5 .** **Genome-wide association results for imputed SNPs (p<1×10^-4^ in combined analysis) in the vicinity of *CXCR4* in our JIA cohort.**

| **CHR** | **SNP** | **BP^A^** | **Type** | **Minor/Major Alleles^A^** | **Case MAF** | **Control MAF** | **p value (Discovery)** | **OR^B^** | **p value (Combined Meta-analysis)** |
| --- | --- | --- | --- | --- | --- | --- | --- | --- | --- |
| 2 | rs6716987 | 136679964 | Imputed | A/C | 0.273 | 0.390 | 4.86E-10 | 0.59 | 1.38E-10 |
| 2 | rs4954577 | 136665181 | Imputed | A | 0.274 | 0.390 | 5.99E-10 | 0.59 | 1.78E-10 |
| 2 | rs1519527 | 136684887 | Imputed | A/G | 0.150 | 0.239 | 3.90E-08 | 0.56 | 2.74E-08 |
| 2 | rs4954579 | 136684945 | Imputed | A/G | 0.150 | 0.239 | 4.04E-08 | 0.56 | 2.80E-08 |
| 2 | rs13024450 | 136731081 | Imputed | C/T | 0.394 | 0.504 | 1.17E-08 | 0.64 | 6.98E-08 |
| 2 | rs4452212 | 136732461 | Imputed | G/A | 0.394 | 0.504 | 1.17E-08 | 0.64 | 7.15E-08 |
| 2 | rs13004902 | 136744150 | Imputed | A/G | 0.394 | 0.504 | 1.17E-08 | 0.64 | 7.52E-08 |
| 2 | rs12691881 | 136746138 | Imputed | A/G | 0.394 | 0.504 | 1.24E-08 | 0.64 | 8.00E-08 |
| 2 | rs13018756 | 136724705 | Imputed | C/T | 0.394 | 0.502 | 2.41E-08 | 0.65 | 1.23E-07 |
| 2 | rs4072435 | 136730371 | Imputed | C/T | 0.394 | 0.502 | 2.13E-08 | 0.64 | 1.45E-07 |
| 2 | rs11674937 | 136650999 | Imputed | C/G | 0.174 | 0.264 | 1.38E-07 | 0.59 | 9.15E-07 |
| 2 | rs9973445 | 136595086 | Imputed | C/G | 0.084 | 0.126 | 8.61E-04 | 0.64 | 5.23E-06 |
| 2 | rs12615624 | 136438073 | Imputed | G/A | 0.262 | 0.331 | 1.48E-04 | 0.72 | 9.90E-06 |
| 2 | rs745500 | 136299662 | Imputed | G/A | 0.269 | 0.340 | 9.28E-05 | 0.71 | 1.62E-05 |
| 2 | rs7579771 | 136296730 | Imputed | A/T | 0.269 | 0.340 | 8.49E-05 | 0.71 | 1.66E-05 |
| 2 | rs3754686 | 136319746 | Imputed | T/C | 0.268 | 0.341 | 6.36E-05 | 0.71 | 1.83E-05 |
| 2 | rs3769005 | 136319836 | Imputed | C/G | 0.268 | 0.341 | 6.36E-05 | 0.71 | 1.83E-05 |
| 2 | rs4954490 | 136324701 | Imputed | G/A | 0.268 | 0.341 | 6.65E-05 | 0.71 | 1.87E-05 |
| 2 | rs892715 | 136293047 | Imputed | T/C | 0.269 | 0.340 | 8.74E-05 | 0.71 | 2.22E-05 |
| 2 | rs1435576 | 136358352 | Imputed | T/A | 0.274 | 0.352 | 2.73E-05 | 0.69 | 2.42E-05 |
| 2 | rs309125 | 136360025 | Imputed | T/C | 0.274 | 0.352 | 2.86E-05 | 0.69 | 2.44E-05 |
| 2 | rs7589832 | 136220571 | Imputed | C/A | 0.194 | 0.256 | 1.90E-04 | 0.70 | 4.25E-05 |
| 2 | rs632632 | 136354686 | Imputed | C/T | 0.274 | 0.350 | 4.67E-05 | 0.70 | 4.85E-05 |
| 2 | rs12619365 | 136114644 | Imputed | T/C | 0.258 | 0.324 | 2.36E-04 | 0.73 | 6.02E-05 |
| 2 | rs2839740 | 136365353 | Imputed | T/G | 0.158 | 0.219 | 9.84E-05 | 0.67 | 6.51E-05 |
| 2 | rs7581814 | 136358063 | Imputed | G/C | 0.158 | 0.219 | 9.64E-05 | 0.67 | 6.57E-05 |

BP, base pair chromosome coordinates; MAF, minor allele frequencies in discovery cohort; p value, basic allelic test p value; OR, odds ratio in discovery cohort; p value (combined), meta-analysis p value in the three cohorts typed genome-wide (discovery and replication cohorts 1 and 2).

^A^The chromosome coordinates and allele designations are on the basis of the forward strand of the NCBI 36 genome assembly.

^B^The odds ratio is calculated with respect to the minor allele.

**Supplemental Table 6. Primers used in Sanger sequencing validation of rare variants at *CXCR4* locus.**

| **Chr** | **Pos** | **Primers** | |
| --- | --- | --- | --- |
|  |  | **Forward** | **Reverse** |
| Chr2 | 136872705 | GATGAATGTCCACCTCGCTT | CTCCAAGCTGTCACACTCCA |
| Chr2 | 136872970 | TGGAGTGTGACAGCTTGGAG | GGCAAACTGGTACTTTGGGA |
| Chr2 | 136873341 | TCCCAAAGTACCAGTTTGCC | TGCTTGCTGAATTGGAAGTG |
| Chr2 | 136873491 | TTGTCCGTCATGCTTCTCAG | GGAAAAGATGGGGAGGAGAG |
| Chr2 | 136873496 | TTGTCCGTCATGCTTCTCAG | GGAAAAGATGGGGAGGAGAG |

Chr: chromosome; Pos: position on human genome build hg19.

**Supplemental Figure 1.** **Genome-wide association results for JIA.**

Manhattan plot showing the association statistics of SNPs from the GWAS of the discovery cohort. The SNP positions are indicated on the X-axis and the -log10(p values) of each SNP is shown on the Y-axis. P values down to 10^-20^ only are shown for optimal resolution; p values for the *HLA* locus on chromosome 6 reached 2.63x10^-23^.

**Supplemental Figure 2. Regional association plot for the 2q22.1 region.**

Genotyped (triangle) and imputed (circles) SNPs are plotted with their combined p values in the three cohorts typed genome-wide (discovery and replication cohorts 1 and 2). SNPs are colored on the basis of their correlation with rs953387 (red: *r*^2^≥0.8; orange: 0.5≤*r*^2^<0.8; yellow: 0.2≤*r*^2^<0.5). Estimated recombination rates from HapMap data are plotted to reflect the local linkage disequilibrium (LD) structure.

**Supplemental Figure 3.** ***CXCR4* tissue-specific gene expression levels** (probe identifiers: 217028_at,

211919_s_at, and 209201_x_at), based on the GNF SymAtlas database on 79 human tissues. Expression of *CXCR4* is most prominent in the CD-annotated T-cells, B-cells, NK and dendritic cells.


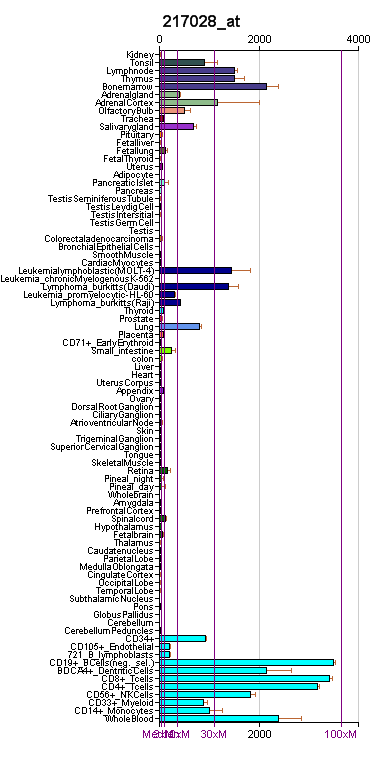

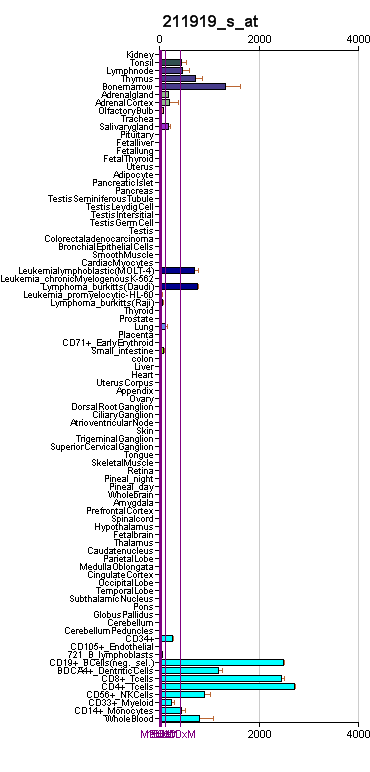

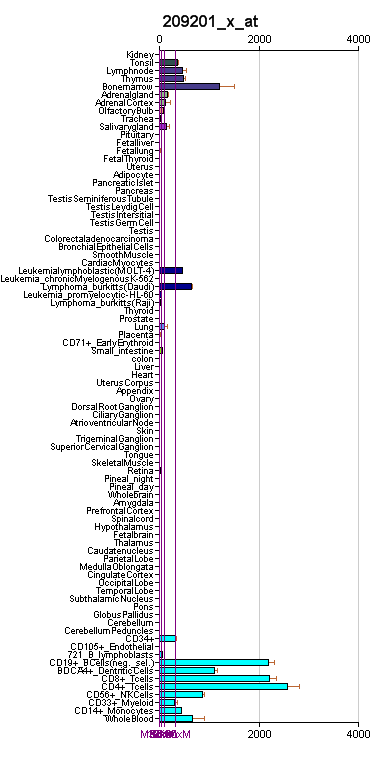


**Supplemental Figure 4. CXCR4 expression levels stratified by SNP genotype.**

The SNP genotypes of rs953387 and rs1016269 are associated with *CXCR4* transcript levels by quantitative PCR (eQTL) in immortalized B-cell lines (from 30 CEU children) and T-cells (from umbilical cords of 75 individuals of Western European origin), respectively.

**Supplemental References**

1. Petty RE et al. International League of Associations for Rheumatology classification of juvenile idiopathic arthritis: second revision, Edmonton, 2001. *J Rheumatol*. 2004;31(2):390–392.
